# Real-world data on ranibizumab for myopic choroidal neovascularization due to pathologic myopia: results from a post-marketing surveillance in Japan

**DOI:** 10.1038/s41433-018-0192-2

**Published:** 2018-08-29

**Authors:** Kyoko Ohno-Matsui, Makoto Suzaki, Rie Teshima, Nina Okami

**Affiliations:** 1 Department of Ophthalmology and Visual Science, Tokyo Medical and Dental University, Tokyo, Japan; 2 Medical Scientific Expert, Medical Division, Novartis Pharma K.K., Tokyo, Japan; 3 Biostatistics, Japan Development, Novartis Pharma K.K., Tokyo, Japan; 4 Patient Safety Japan, Regulatory Office Japan, Novartis Pharma K.K., Tokyo, Japan

## Abstract

**Objectives:**

The aim of this study was to obtain real-world clinical data on the safety and efficacy of ranibizumab treatment for myopic choroidal neovascularization (CNV) due to pathologic myopia.

**Methods:**

This was a prospective, observational, post-marketing surveillance study in ranibizumab-naive Japanese patients with myopic CNV. Patients who initiated ranibizumab treatment were registered and prospectively observed over 12 months. Safety endpoints were the incidence of ocular and non-ocular adverse drug reactions (ADRs) and serious adverse events (SAEs). The efficacy endpoint included the average change in best-corrected visual acuity (BCVA) in logarithm of the minimal angle of resolution (logMAR) units (logMAR BCVA) from baseline to the last observation.

**Results:**

Three hundred and eighteen patients were included in the safety analysis population. Of these 79.9% were female and the mean age was 65.5 years. The incidences of SAEs and ADRs were 0.6 and 0.3%, respectively. A total of 268 patients (84.0%) completed the 12-month study period. The mean (±SD) and median number of ranibizumab injections were 2.0 ± 1.5 and 1.0 during the 12-month study period, respectively. The number of ranibizumab injections received was one in 52.2% of patients and less than or equal to three in 89.2%. The mean change in logMAR BCVA from baseline to month 12 was −0.193, and the mean logMAR BCVA improved from 0.517 to 0.319 between baseline and month 12.

**Conclusions:**

This study showed that ranibizumab is generally well tolerated, and that a minimum number of injections were necessary to produce a therapeutic effect in Japanese myopic CNV patients in a real-world setting.

## Introduction

Pathologic myopia is a degenerative eye condition characterized by an abnormally elongated axial length that is ≥3 standard deviations longer than the axial length of an emmetropic eye [1, 2]. Axial elongation causes the optical components of the eye to focus the image in front of the retina, and this is known as myopia. Myopia is defined by the refractive error of the lens and is measured in diopters (D). High myopia is characterized by values exceeding −6.0 D and pathologic myopia is characterized by values greater  than −8.0 D [3].

The biomechanical forces related to axial elongation also cause progressive retinochoroidal thinning, which can lead to profound vision loss. Pathologic myopia can also cause a range of pathologically morphological changes in the posterior fundus, notably choroidal neovascularization (CNV). Pathologic myopia is relatively common in Asian populations compared with non-Asian populations [4]. For example, in the Hisayama study, the prevalence of pathologic myopia (myopic retinopathy) in Japanese patients aged over 40 was 1.2% in men and 2.2% in women [5].

A recent retrospective chart review study conducted in Japan has also revealed significant increases in the axial length in highly myopic eyes, with CNV eyes showing even greater increases [6]. Myopic CNV occurs in approximately 10% of patients with high myopia, and one-third of those affected have CNV in both eyes [7]. The visual prognosis in myopic CNV patients is poor and, if left untreated, the disease decimates visual acuity (VA), resulting in ≤0.1 VA in 89% of patients after 5 years and in 96% of patients after 10 years [8].

Although the mechanism behind CNV in pathologic myopia remains unclear, vascular endothelial growth factor (VEGF) has been shown to be present in high concentrations in the aqueous humor in myopic CNV eyes [9]. At present, anti-VEGF medications have produced a therapeutic effect in several clinical studies [10–12]. In particular, the phase III RADIANCE study compared ranibizumab, the Fab fragment of anti-VEGF monoclonal antibody, with verteporfin photodynamic therapy in myopic CNV patients and found that ranibizumab produces superior best-corrected VA (BCVA) gains that were maintained for 1 year [10].

Based on the results from the RADIANCE study, ranibizumab has been approved for the treatment of myopic CNV due to pathologic myopia (myopic CNV) in many countries throughout Europe and Asia. These findings led us to consider that an anti-VEGF treatment could be the first-line therapy, and that the treatment should be initiated promptly once myopic CNV was diagnosed using fluorescein angiography [13, 14]. A retrospective cohort study using the Intelligent Research in Sight (IRIS) Registry in the US reported the efficacy of anti-VEGF treatment, including ranibizumab, for treatment-naive myopic CNV patients [15]. However, at present, there are insufficient safety and efficacy data for ranibizumab in Japanese patients with myopic CNV. The RADIANCE study enrolled only 50 Japanese patients [10], and there is a lack of real-world clinical setting efficacy and safety data in Japanese and other Asian populations [16–19]. In this study, we obtained real-world data on the safety and efficacy of ranibizumab in Japanese patients with myopic CNV.

## Subjects and methods

### Study design

This study was a prospective, 12-month, multicenter, observational, post-marketing surveillance conducted at 73 sites in Japan. The purpose of this study was to evaluate the safety and effectiveness of ranibizumab in Japanese patients with myopic CNV. This study was conducted in accordance with the Good Post-Marketing Study Practice.

Patients were enrolled between February 2014 and February 2016. For this interim analysis, the cut-off date for the receipt of case report forms (CRFs) was 5 October 2017. The observational period was 12 months after the first day of ranibizumab treatment.

### Patients

Eligible patients had to be ranibizumab-naive Japanese patients diagnosed with myopic CNV. The investigators at each site made the diagnosis of myopic CNV, but diagnostic criteria such as eye axis and refractive index were not defined in this study. Written informed consent was obtained prior to the start of ranibizumab treatment.

Patients were enrolled by Day 14, via an Electronic Data Capture (EDC) system, with the first day of ranibizumab treatment designated as Day 1. Patients were not eligible for enrollment to the study if they had previously been treated with ranibizumab in either eye. However, previous treatment with other anti-VEGF agents was not a disqualifying factor.

### Observational data collection

In this observational study, patient characteristics, such as sex, age, reason for treatment (including which eye), first day of treatment, pregnancy status, treatment classification, height, body weight, BMI, duration of myopic CNV, past medical history, complications, presence or absence of history of high myopia and its disease duration, habitual drinking status, habitual smoking status, and predisposing factors for hypersensitivity, were recorded.

Details on concomitant therapies for myopic CNV were also recorded. Other patient details also collected during this study included decimal BCVA, intraocular pressure, and central retinal thickness (CRT). In this study, only the data collected on the first day of treatment were included in establishing the baseline for each patient characteristic.

Reasons for treatment discontinuation or dropout were classified as follows: an adverse event (AE) that occurred after ranibizumab administration irrespective of any causal relationship, insufficient therapeutic effect, did not visit after the first day of treatment, stopped visiting, transferred to another medical institution, at the request of patient/family member, patient withdrew consent, or other. Discontinuations and dropout assessments included status of treatment, date of discontinuation or dropout, and reason for discontinuation.

### Treatment

Ranibizumab treatment was administered according to the Japanese medical package insert on dosage and administration route. Ranibizumab at 0.5 mg (0.05 mL) per dose was intravitreally injected, with multiple injections requiring a treatment interval of no less than one month. Patients whose visual impairment resolved or improved after the start of treatment, who therefore no longer required periodic treatment, continued undergoing observation without being discontinued from the study.

Treatment exposure was defined as the number of ranibizumab injections administered over the 12-month observation period from baseline (Day 1).

### Safety evaluation

The safety endpoints evaluated were the incidence of ocular and non-ocular adverse drug reactions (ADRs) and serious AEs (SAEs), the relationship between the presence or absence of ocular ADRs and patient factors, and the relationship between the presence or absence of non-ocular ADRs and patient factors. An AE was defined as any undesirable event occurring in a patient after administration of ranibizumab, regardless of whether it was considered to be related to ranibizumab or not. An ADR was defined as an AE for which a causal relationship with ranibizumab could not be ruled out or an AE attributed to the intravitreal injection procedure.

Safety variables included the incidence of AEs. AEs were classified using the Medical Dictionary for Regulatory Activities/Japanese edition (MedDRA/J) version 20.0.

### Efficacy evaluation

The key efficacy endpoint was the average change in BCVA in logarithm of the minimal angle of resolution (logMAR) units (logMAR BCVA) from baseline (Day 1) to the last observation date when the last decimal VA exam was performed. Baseline BCVA was measured on the day of the first intravitreal injection, prior to the injection. Efficacy was graded based on average changes in logMAR BCVA: average change in logMAR BCVA ≤−0.3 was defined as “improved”, average change in logMAR BCVA >−0.3 and <0.3 was defined as “stable”, and average change in logMAR BCVA ≥0.3 was defined as “deteriorated”. Collectively, “improved” and “stable” were defined as “effective” in this study. In addition, changes in logMAR BCVA over time were examined using changes in logMAR BCVA from baseline logMAR BCVA and absolute logMAR BCVA. These changes over time were categorized by baseline decimal BCVA (≥0.6, 0.3–<0.6, <0.3).

Other efficacy endpoints were the proportions graded as “effective” categorized by patient demographic characteristics, such as sex, age, reason for treatment, duration of myopic CNV, complications, past medical history, BMI, habitual drinking status, habitual smoking status, concomitant drugs at baseline, and baseline BCVA.

### Statistical analysis

In terms of the sample size, we decided to conduct a study targeting 300 cases, which is the number of cases that could confirm one or more AEs with an occurrence rate of ≥1% and a probability of at least 95%.

Safety was analyzed using the safety analysis population, which consisted of patients with fixed CRF data, except for (1) those who were previously treated with ranibizumab, and (2) those who were registered in the EDC system after receiving ranibizumab for ≥14 days. Efficacy was analyzed using the efficacy analysis population, which excluded patients with missing data on VA measured at baseline and/or after ranibizumab treatment.

Patients were centrally enrolled, and their data were collected. When analyzing the relationship between each efficacy endpoint and patient factors, Fisher’s exact test was used for factors measured at the nominal level, and the Mann–Whitney U-test was used for factors measured at the ordinal level.

When examining changes in logMAR BCVA over time, the mean and SD values were calculated in the patient population with a decimal BCVA measured at baseline and after the start of ranibizumab treatment at each time point.

The statistical analysis software used was SAS 9.3 (SAS Institute Inc., Cary, NC). The construction, management, and operation of the EDC system, patient enrollment, progress management, data management, and analytical and tabulation duties were outsourced to Asklep, Inc. (Tokyo, Japan).

## Results

### Patient characteristics

**Fig. 1:**
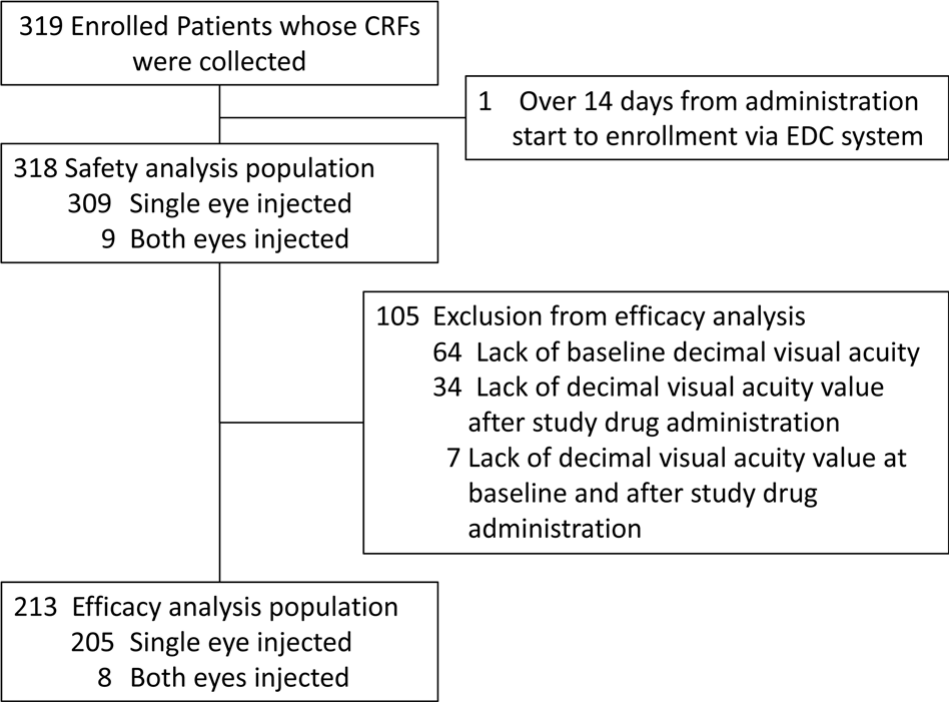
Patient disposition. CRF case report form, EDC electronic data capture, VA visual acuity

In this study, 319 patients whose CRFs were collected after they were examined for eligibility were enrolled (Fig. 1). The safety analysis population included 318 patients, of whom 309 had one eye treated, and 9 had both eyes treated. For patients with both eyes treated, only the eye with the earlier treatment start date was included in the analyses.

One hundred and five patients were excluded from the efficacy analysis population due to a lack of baseline decimal BCVA (*n* = 64), lack of decimal BCVA value after study drug administration (*n* = 34), and lack of decimal BCVA value at baseline and after study drug administration (*n* = 7). This left 213 patients in the efficacy analysis population, of whom 205 had one eye treated, and 8 had both eyes treated. For patients with both eyes treated, only the eye with the earlier treatment start date was included in the analyses (*n* = 213 eyes).

The mean observation period (SD) was 326.4 (87.9) days in the safety analysis population, and the demographic and clinical characteristics are shown in Table 1.

**Table 1:** Patient characteristics

Characteristics	Safety analysis population (*n* = 318)	Efficacy analysis population (*n* = 213)
Mean age, years (SD)	65.6 (13.3)	66.7 (13.0)
≥50 years	279 (87.7)	191 (89.7)
≥65 years	196 (61.6)	138 (64.8)
Sex		
Male	64 (20.1)	39 (18.3)
Female	254 (79.9)	174 (81.7)
Pregnant	0	0
Duration of disease		
Number of patients^a^	92	79
Months, mean (SD)	9.9 (34.5)	9.5 (36.5)
LogMAR BCVA, mean (SD)	0.50 (0.40)	0.50 (0.40)
Mean decimal BCVA		
≥0.6 (logMAR conversion ≤0.22)	81 (25.5)	68 (31.9)
≥0.3−<0.6 (logMAR conversion >0.22−≤0.52)	76 (23.9)	68 (31.9)
<0.3 (logMAR conversion >0.52)	90 (28.3)	77 (36.2)
NA, *n* (%)	71 (22.3)	0 (0.0)
Complications		
Impaired liver function	7 (2.2)	7 (3.3)
Renal impairment	2 (0.6)	1 (0.5)
Glaucoma	27 (8.5)	19 (8.9)
Ocular hypertension	1 (0.3)	1 (0.5)
Stroke	1 (0.3)	1 (0.5)
Medical history	111 (34.9)	86 (40.4)
Concomitant drugs	49 (15.4)	41 (19.3)

*BCVA* best-corrected visual acuity, *CNV* choroidal neovascularization, *CRT* central retinal thickness, *IOP* intraocular pressure, *NA* not assessed, *SD* standard deviation, *VA* visual acuity

Number of patients (%), unless otherwise stated

^a^Case with a record of disease duration

In the safety analysis population, the percentages of males and females were 20.1 and 79.9%, respectively. The mean age was 65.5 years with the percentages of patients aged ≥50 years and ≥65 years being 87.7 and 61.6%, respectively. The percentage of patients with comorbid glaucoma was 8.5%. The percentage of patients using concomitant drugs at baseline was 15.4%.

In the efficacy analysis population, the percentages of males and females were 18.3 and 81.7%, respectively, and the mean age was 66.7 years. These population characteristics were similar to those in the safety analysis population.

### Safety

In the safety analysis population, one patient (0.3%) had an ocular ADR during the observational period. No non-ocular ADRs were observed (Table 2). One case of vitreous hemorrhage was defined as an ADR, but it was non-serious. SAEs occurred in two patients. One patient had one event of optic neuritis, which was reported as an ocular SAE. The other patient had non-ocular SAEs, which were one event each of hepatitis C and hemangioma.

**Table 2:** Adverse events

Preferred term	Total AEs (*n* = 318)	Serious AEs (*n* = 318)	Adverse drug reactions (*n* = 318)
Incidences (number of patients)	11 (3.5)	2 (0.6)	1 (0.3)^a^
Incidences (number of events)	17 (−)	3 (−)	1 (−)^a^
Deaths	0	0	0
Ocular AEs, total	10 (3.1)	1 (0.3)	1 (0.3)^a^
Blepharitis	2 (0.6)	0	0
Blepharospasm	2 (0.6)	0	0
Drug ineffective	2 (0.6)	0	0
Optic neuritis	1 (0.3)	1 (0.3)	0
Vitreous hemorrhage	1 (0.3)	0	1 (0.3)^a^
Cataract	1 (0.3)	0	0
Eyelid ptosis	1 (0.3)	0	0
Tick epithelitis	1 (0.3)	0	0
Choroidal neovascularization	1 (0.3)	0	0
Conjunctivitis	1 (0.3)	0	0
Ocular hypertension	1 (0.3)	0	0
Non-ocular AEs, total	2 (0.6)	1 (0.3)	0
Hepatitis C	1 (0.3)	1 (0.3)	0
Hemangioma	1 (0.3)	1 (0.3)	0
Hypertension	1 (0.3)	0	0

*AE* adverse event, *MedDRA* medical dictionary for regulatory authorities

Number of patients (%)

A patient with multiple occurrences of an AE under one treatment is counted only once in the AE category for that treatment

Adverse events were classified into the preferred term of the MedDRA/J version 20.0

^a^This event was classified as a non-serious AE

Optic neuritis occurred on day 182 after the initial ranibizumab treatment in an 81-year-old woman. It was after the fourth administration of ranibizumab, and the duration from the fourth administration to the onset of the SAE was 21 days. The SAE resolved 14 days after its onset and was deemed not to be related to ranibizumab. No endophthalmitis or deaths were observed. Seventeen AEs, including SAEs, were reported in 11 patients (3.5%). There was no peak in the time of AE onset, regardless of treatment start time (Fig. S1).

### Treatment exposure

**Fig. 2:**
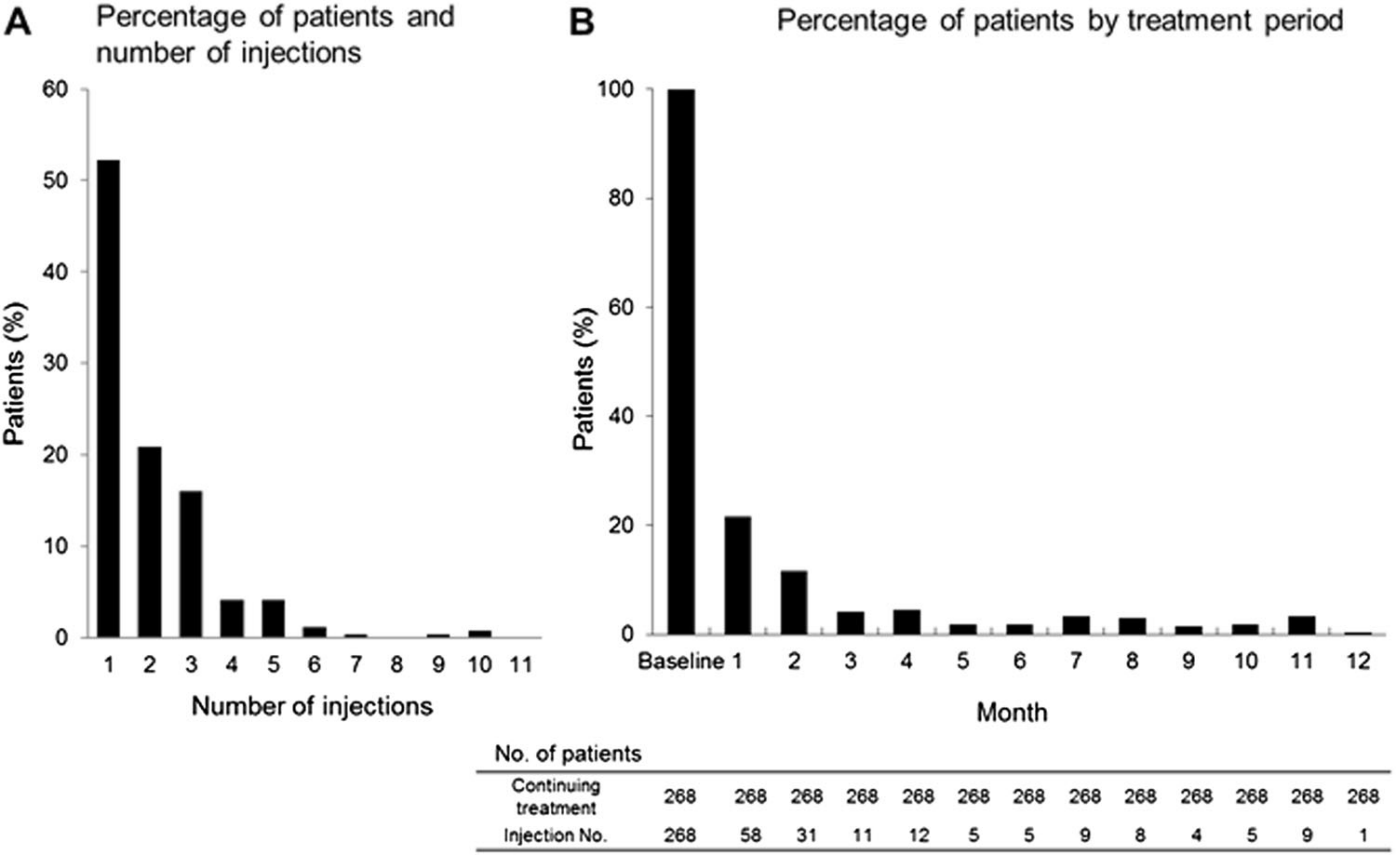
Treatment exposure: percentage of **a** patients and number of injections and **b** patients by treatment period (safety analysis population, *n* = 268)

In the safety analysis population, 268 patients who completed the 12-month observation period were assessed for the number of ranibizumab injections (Fig. 2). During the mean observation period of 360.3 ± 2.5 days, the mean (±SD) and median [min–max] number of ranibizumab injections were 2.0 ± 1.5 and 1.0 [1–10], respectively. One hundred and forty patients (52.2%) received ranibizumab treatment only once, and 239 (89.2%) received treatment ≤3 times during the observation period. There was a similar trend in the distribution of the number of ranibizumab injections between the safety analysis population and the efficacy analysis population.

For each treatment interval during the first 12 months of ranibizumab treatment, the proportion of patients being followed up that were receiving ranibizumab (the retreatment rate) was calculated (Fig. 2). The retreatment rates were 21.6, 11.6, and ≤5% at month 1, month 2, and month 3, respectively, after the start of ranibizumab treatment.

During this study, there were only three patients treated for myopic CNV with a drug or therapy other than ranibizumab (laser photocoagulation, *n* = 1; anti-VEGF agent, *n* = 1; steroid injection, *n* = 1).

### Efficacy

A total of 20.7% of all patients achieved an improvement in VA (average change in logMAR BCVA ≤−0.3), and 22.2% of patients treated once achieved an improvement in VA (Table S1). Moreover, in 95.8% of patients overall and 98.9% of once-treated patients, the efficacy of treatment was graded “effective” (Table S1). Factors related to efficacy were examined and no relationship was found with any patient demographic characteristic (data not shown).

**Fig. 3:**
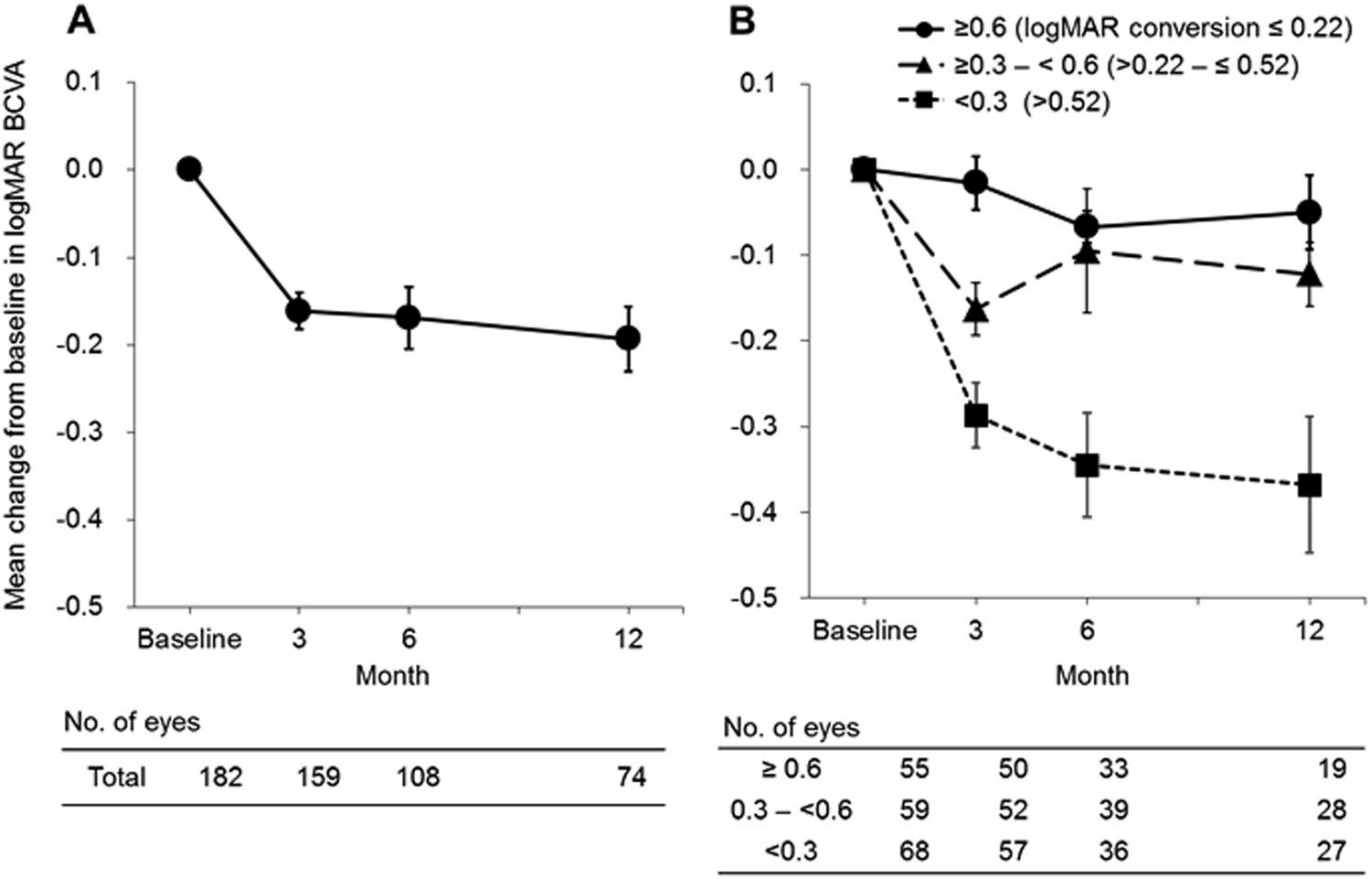
Mean change in logMAR BCVA from baseline to month 12 in subjects with available data at two or more time points (**a** total and **b** categorized by baseline values) (*n* = 182)

The mean changes in logMAR BCVA from baseline to months 3, 6, and 12 were −0.161, −0.169, and −0.193, respectively (Fig. 3a).

The mean logMAR BCVA improved from 0.517 at baseline to 0.319 at month 12. When categorized by baseline decimal BCVA, the low baseline BCVA group showed greater logMAR BCVA improvement than the high baseline BCVA group (Figs. 3b and S2). However, logMAR BCVA values at the end of the observation period were better in the high baseline BCVA group than in the low baseline BCVA group (Fig. S2). There was no correlation between the frequency of ranibizumab injections and the average change in logMAR BCVA from baseline to the last observation (data not shown). The mean changes in CRT from baseline to months 3 and 12 were −72.1 and −106.4, respectively (Fig. S3).

## Discussion

This is the first large-scale, multi-center, observational, prospective study to evaluate the safety and efficacy of ranibizumab for patients with myopic CNV in a real-world setting in Japan. The patients in this study had a mean age of 65.6 years, a logMAR BCVA of 0.50 ± 0.40, and 79.9% were female. Compared with the patients in the RADIANCE study, the patients in this study were slightly older (54–56 years of age) and had slightly better BCVA (55.4–55.8 Early Treatment Diabetic Retinopathy Study (ETDRS) letters: 0.60 logMAR equivalent).

The inclusion criterion for BCVA in the RADIANCE study was 24–78 ETDRS letters (20/320–20/32 Snellen equivalent) [10]. The percentage of patients with a decimal BCVA ≥0.6 in this study was between 25.5 and 31.9%, showing a difference in the baseline BCVA from that in the RADIANCE study.

The primary objective of this study was to provide information on the safety of ranibizumab for patients with myopic CNV in a real-world setting. We found that the incidences of AEs, SAEs, ADRs, and SADRs in this study were 3.4, 0.6, 0.3, and 0%, respectively. In addition, the incidences of ocular AEs and non-ocular AEs in this study were 3.1 and 0.9%, respectively, and were generally lower than those in the RADIANCE study. Of 11 patients with AEs reported in this study, 10 patients were assessed to have AEs that were not related to ranibizumab with the remaining 1 patient identified as having an ADR. Additionally, the time of onset of AEs at each treatment interval after the start of ranibizumab treatment was balanced with respect to the time of AE onset. This suggests that AEs are less likely to be related to ranibizumab treatment.

In the clinical course of myopic CNV treatment, the main concern is the development of macular atrophy, which involves the central fovea [20–22]. Development of chorioretinal atrophy around the regressed CNV is the main reason for visual impairment in eyes with myopic CNV [8]. However, in this study, no macular atrophy was reported as an AE, and only 4.2% of patients were reported to have VA deterioration (logMAR change ≥0.3). In this study, and in the RADIANCE study, there were no deaths and no cases of endophthalmitis, retinal detachment, myocardial infarction, or cerebrovascular events [10].

Over the course of the 12-month observation period, in 95.8% of all patients, the efficacy of treatment was graded “effective” after an average of two ranibizumab injections. This rate was comparable to that in the RADIANCE study (98.1–99.1%). This suggested that ranibizumab treatment in myopic CNV patients was effective at improving and maintaining VA, even with a small number of injections, in real-world clinical settings. This study also showed that the improvement of logMAR BCVA at 12 months was 0.193, which corresponds to 9.65 ETDRS letters. This ETDRS score is slightly lower compared with the values from the RADIANCE study (13.8–14.4 letters). This difference is possibly due to the following reasons: the baseline VA in this study was better than that in the RADIANCE study; and the mean number of ranibizumab injections over a 12-month period in the RADIANCE study was 4.6 in the group in which administration was guided by VA stabilization criteria, and 3.5 in the group in which administration was guided by disease activity criteria, both values being higher than those reported in this study.

In this study, an improvement of 0.193 in logMAR BCVA was observed over 12 months of treatment in the efficacy analysis population. In comparison, the IRIS Registry had a comparable baseline VA and the extent of improvement in VA (0.17 units) with anti-VEGF treatment was also similar. However, the mean number of ranibizumab injections in this study was 2.0 ± 1.5, which was lower than that in the IRIS Registry (2.8 ± 2.5) [15]. A subgroup analysis of the RADIANCE study reported that the median number of ranibizumab injections over 12 months was lower in East Asian patients (median = 2) than in Caucasian patients (median = 3) [23]. This suggests that ethnic differences may influence the number of ranibizumab injections required, although the effect of ranibizumab on VA was similar in these studies.

The RADIANCE subgroup analysis showed that the mean gain in BCVA tended to be higher in patients with lower baseline BCVA [23]. A similar trend was also observed in this study. Therefore, ranibizumab treatment appears to also be useful in routine clinical practice in Japan for patients with low VA.

On the other hand, the absolute BCVA values at the last observation visit were higher in patients with higher baseline BCVA values in this study. This shows that starting treatment at an earlier stage of VA decline allows patients to maintain better VA. According to the Consensus Statement, early intervention is recommended for myopic CNV [14]. This is because in a previous 10-year follow-up study, the long-term prognosis for patients with myopic CNV was found to be very poor if left untreated [8]. This study also demonstrates that patients with a high VA should start ranibizumab treatment at an early stage as this can lead to the maintenance of high VA.

Although favorable safety and efficacy profiles were observed over the 12-month period, an extended follow-up is needed to demonstrate the long-term safety and efficacy of ranibizumab, as was shown during the 5-year follow-up of the RADIANCE clinical trial [24]. An unresolved problem with long-term therapy for myopic CNV is decreased VA from CNV-related macular atrophy following successful CNV resolution [20, 21, 25]. Further investigation is needed to determine whether long-term treatment with ranibizumab causes similar problems.

The potential limitations of this study include the subjective diagnosis of myopic CNV by an attending physician under a routine practice, which therefore may be different from the standard diagnostic criteria. Another limitation is the absence of patient data on axial length, refractive index, and concomitant therapy as these were not included in the preplanned statistical analysis plan. Furthermore, collection of baseline data on VA were only allowed on Day 1. Because this was an observational study, some of the clinical practice physicians did not assess VA on Day 1 of the study, which resulted in a higher than expected exclusion rate. However, this was unlikely to induce any bias to the study results.

In conclusion, this study demonstrated that ranibizumab treatment in Japanese myopic CNV patients has a tolerable safety profile and produces a therapeutic effect with a small number of injections in a real-world clinical setting. For patients who maintained high BCVA values at the end of treatment, it is surmised that ranibizumab treatment should be started as soon as myopic CNV is diagnosed.

### Summary

#### What was known before

- Myopic choroidal neovascularization (CNV) occurs in ~10% of patients with high myopia, and one-third of those affected have CNV in both eyes.
- Although the mechanism behind CNV in pathologic myopia remains unclear, vascular endothelial growth factor (VEGF) has been shown to be present in high concentrations in the aqueous humor in myopic CNV eyes.
- The phase III RADIANCE study showed that ranibizumab, the Fab fragment of anti-VEGF monoclonal antibody, produced visual acuity gains that were maintained for 1 year, but only enrolled 50 Japanese patients; thus, there are insufficient safety and efficacy data for ranibizumab in Japanese patients with myopic CNV due to pathologic myopia.

#### What this study adds

- We obtained real-world data on the safety and efficacy of ranibizumab injections in Japanese patients with myopic CNV due to pathologic myopia.
- The mean change in logMAR best-corrected visual acuity from baseline to month 12 was −0.193, after 2.0 ± 1.5 (mean ± SD; median of 1.0) ranibizumab injections; 89.2% receiving treatment ≤3 times during the observation period.
- Our findings show that ranibizumab is generally well tolerated, and that a minimum number of injections are necessary to produce a therapeutic effect in Japanese myopic CNV patients in a real-world setting.

## Electronic supplementary material

Supplementary Legends

Supplementary Table

Supplementary Figure 1

Supplementary Figure 2

Supplementary Figure 3
