# Supplementary material for: Real-world data on ranibizumab for myopic choroidal neovascularization due to pathologic myopia: results from a post-marketing surveillance in Japan
Source: Eye (Lond). 2018 Aug 29;32(12):1871–8. doi: 10.1038/s41433-018-0192-2 (PMC6292850; doi:10.1038/s41433-018-0192-2)
Supplement: Supplementary file 1 — Supplementary Legends [file 41433_2018_192_MOESM1_ESM.docx]

**Titles and legends to supplementary table and figures**

**Table S1** Ranibizumab treatment exposure and average change in logMAR BCVA.

Abbreviation: VA, visual acuity.

**Figure S1** Incidence of adverse events by treatment period.

**Figure** **S2** Mean logMAR BCVA from baseline to month 12 ((A) total and (B) categorized by baseline BCVA).

**Figure S3** Mean change in central retinal thickness (CRT) from baseline up to month 12.
