# Supplementary material for: Real-world data on ranibizumab for myopic choroidal neovascularization due to pathologic myopia: results from a post-marketing surveillance in Japan
Source: Eye (Lond). 2018 Aug 29;32(12):1871–8. doi: 10.1038/s41433-018-0192-2 (PMC6292850; doi:10.1038/s41433-018-0192-2)
Supplement: Supplementary file 2 — Supplementary Table [file 41433_2018_192_MOESM2_ESM.docx]

| Frequency of injection | Total no. of eyes (%) | Mean change in logMAR BCVA mean (SD) | Improvement/deterioration in logMAR BCVA | | | | | | | |
| --- | --- | --- | --- | --- | --- | --- | --- | --- | --- | --- |
|  |  |  | Eyes (%) | | | | | | | |
|  |  |  | ≤−0.3 | | >−0.3–<0.3 | | ≥0.3 | | <0.3 | |
|  |  |  | (Improved) | | (Stable) | | (Deteriorated) | | (Effective) | |
| All patients | 213 (100) | −0.13 (0.29) | 44 | 20.7 | 160 | 75.1 | 9 | 4.2 | 204 | 95.8 |
| 1 | 90 (42.3) | −0.16 (0.30) | 20 | 22.2 | 69 | 76.7 | 1 | 1.1 | 89 | 98.9 |
| 2 | 49 (23.0) | −0.07 (0.32) | 10 | 20.4 | 35 | 71.4 | 4 | 8.2 | 45 | 91.8 |
| 3 | 40 (18.8) | −0.12 (0.25) | 7 | 17.5 | 31 | 77.5 | 2 | 5.0 | 38 | 95.0 |
| 4 | 15 (7.0) | −0.11 (0.37) | 3 | 20.0 | 10 | 66.7 | 2 | 13.3 | 13 | 86.7 |
| 5 | 11 (5.2) | −0.15 (0.26) | 2 | 18.2 | 9 | 81.8 | 0 | 0.0 | 11 | 100.0 |
| 6 | 3 (1.4) | −0.38 (0.15) | 2 | 66.7 | 1 | 33.3 | 0 | 0.0 | 3 | 100.0 |
| 7 | 1 (0.5) | −0.13 ( - ) | 0 | 0.0 | 1 | 100.0 | 0 | 0.0 | 1 | 100.0 |
| 9 | 2 (0.9) | −0.11 (0.01) | 0 | 0.0 | 2 | 100.0 | 0 | 0.0 | 2 | 100.0 |
| 10 | 2 (0.9) | −0.12 (0.10) | 0 | 0.0 | 2 | 100.0 | 0 | 0.0 | 2 | 100.0 |
