# Supplementary figures and images for: Real-world data on ranibizumab for myopic choroidal neovascularization due to pathologic myopia: results from a post-marketing surveillance in Japan
Source: Eye (Lond). 2018 Aug 29;32(12):1871–8. doi: 10.1038/s41433-018-0192-2 (PMC6292850; doi:10.1038/s41433-018-0192-2)

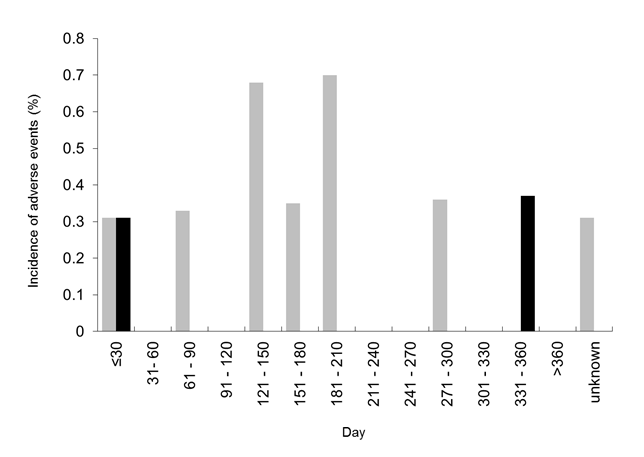

Supplement: Supplementary file 3 — Supplementary Figure 1 [file 41433_2018_192_MOESM3_ESM.tif]

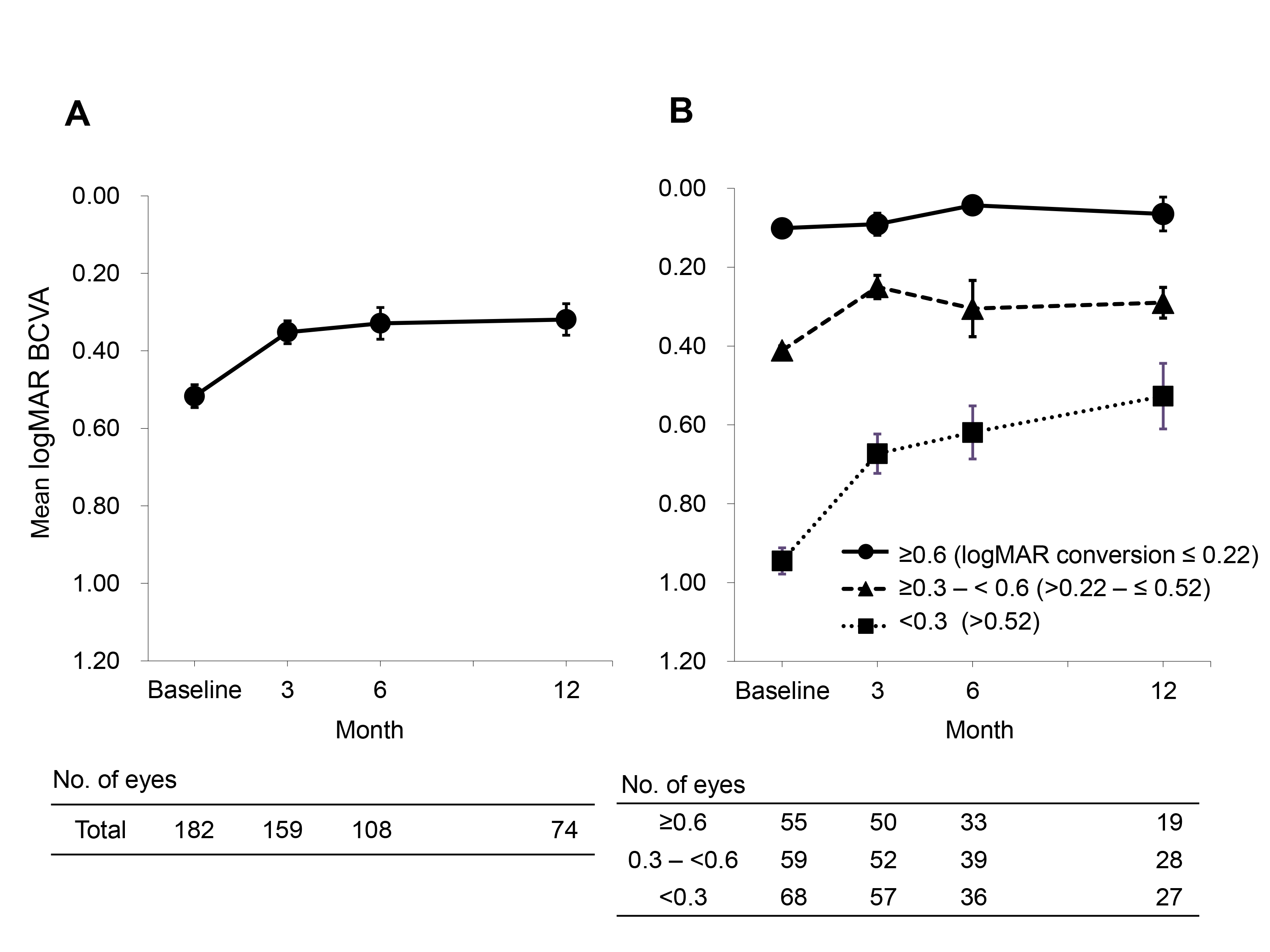

Supplement: Supplementary file 4 — Supplementary Figure 2 [file 41433_2018_192_MOESM4_ESM.tif]

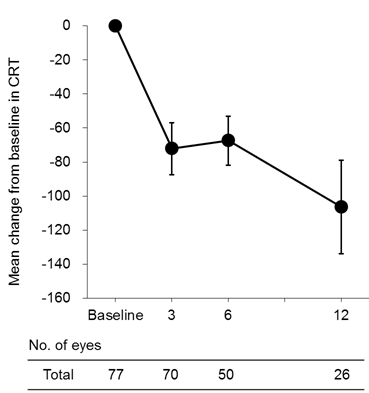

Supplement: Supplementary file 5 — Supplementary Figure 3 [file 41433_2018_192_MOESM5_ESM.tif]
